# Assessment of Weekly Fluctuations in Zarit Burden Interview Scores in Caregivers of Individuals with Cognitive Impairment

**DOI:** 10.1002/alz.092292

**Published:** 2025-01-09

**Authors:** Neil W. Thomas, Bahareh Chimehi, Julien Lariviere‐Chartier, Laura Ault, Bruce Wallace, Zachary T Beattie, Lisa Sheehy, Joel S. Steele, Lyndsey M. Miller

**Affiliations:** ^1^ AGE‐WELL NIH SAM3, Ottawa, ON Canada; ^2^ University of Ottawa, Ottawa, ON Canada; ^3^ Bruyere Research Institute, Ottawa, ON Canada; ^4^ Carleton University, Ottawa, ON Canada; ^5^ NIA‐Layton Aging & Alzheimer’s Disease Research Center, Portland, OR USA; ^6^ Oregon Health & Science University, Portland, OR USA; ^7^ Oregon Center for Aging & Technology (ORCATECH), Portland, OR USA; ^8^ University of North Dakota, Grand Forks, ND USA

## Abstract

**Background:**

Current tools to assess caregiver burden in individuals caring for people living with dementia commonly involve the use of questionnaires administered at infrequent intervals. Caregiver burden can vary significantly between individuals and at different time points throughout the progression of the disease. There has been research investigating the determinants of caregiver burden, but less is known about what represents a clinically‐meaningful amount of intra‐individual change in caregiver burden. The objective of this preliminary analysis is to present data showing change in caregiver burden over time in a cohort with frequent collection of burden level.

**Method:**

Data were derived from longitudinal studies involving the ORCATECH technology platform, consisting of ambient, wearable and other sensors deployed in participants’ homes collecting continuous data on daily activities. Participants were individuals with mild cognitive impairment (MCI) or dementia living with a caregiver (one dyad per home). Caregivers completed the Zarit Burden Interview Short Version (ZBI‐12, range 0‐48) weekly through the duration of their participation. ZBI‐12 scores and their distribution were analyzed.

**Result:**

Data are presented from 47 dyads. Caregivers completed a total of 2949 weekly ZBI‐12 questionnaires (range 2‐118). Caregivers had a mean age of 71.3 and 68% were female. The mean ZBI‐12 score was 15.5 (SD 9.5, range 0‐44). The modal distribution of ZBI‐12 scores demonstrated peaks at 2‐4, 11‐12, and 22‐24, at low, moderate and high levels of burden. The variability in ZBI‐12 scores (standard deviation) increased with the total mean ZBI‐12 score (r^2^ = 0.23) per participant.

**Conclusion:**

Caregiver burden ranged from very low to high and fluctuated week‐to‐week in the majority of caregivers of individuals with MCI and dementia. Caregivers who had a higher average level of burden also had more variability in their ZBI‐12 scores. More frequent assessments of caregiver burden could help to provide a more complete picture of the dementia care situation. Novel approaches, such as continuous monitoring using home sensors, may provide a method to assess variability in burden level. Future work will evaluate changes in ZBI‐12 scores that represent clinically meaningful differences in caregiver burden and sensor outcome measures that predict higher levels of burden.

